# Supplementary material for: Improving yield and fruit quality traits in sweet passion fruit: Evidence for genotype by environment interaction and selection of promising genotypes
Source: PLoS One. 2020 May 14;15(5):e0232818. doi: 10.1371/journal.pone.0232818 (PMC7224466; doi:10.1371/journal.pone.0232818)
Supplement: S1 Fig — Note the difference in the thickness of skin and pulp content between fruits of the parental accessions (above). The six superior genotypes (below) were selected on the basis of a multiplicative index (MI). Bar = 1 cm. (PDF) [file pone.0232818.s004.pdf]

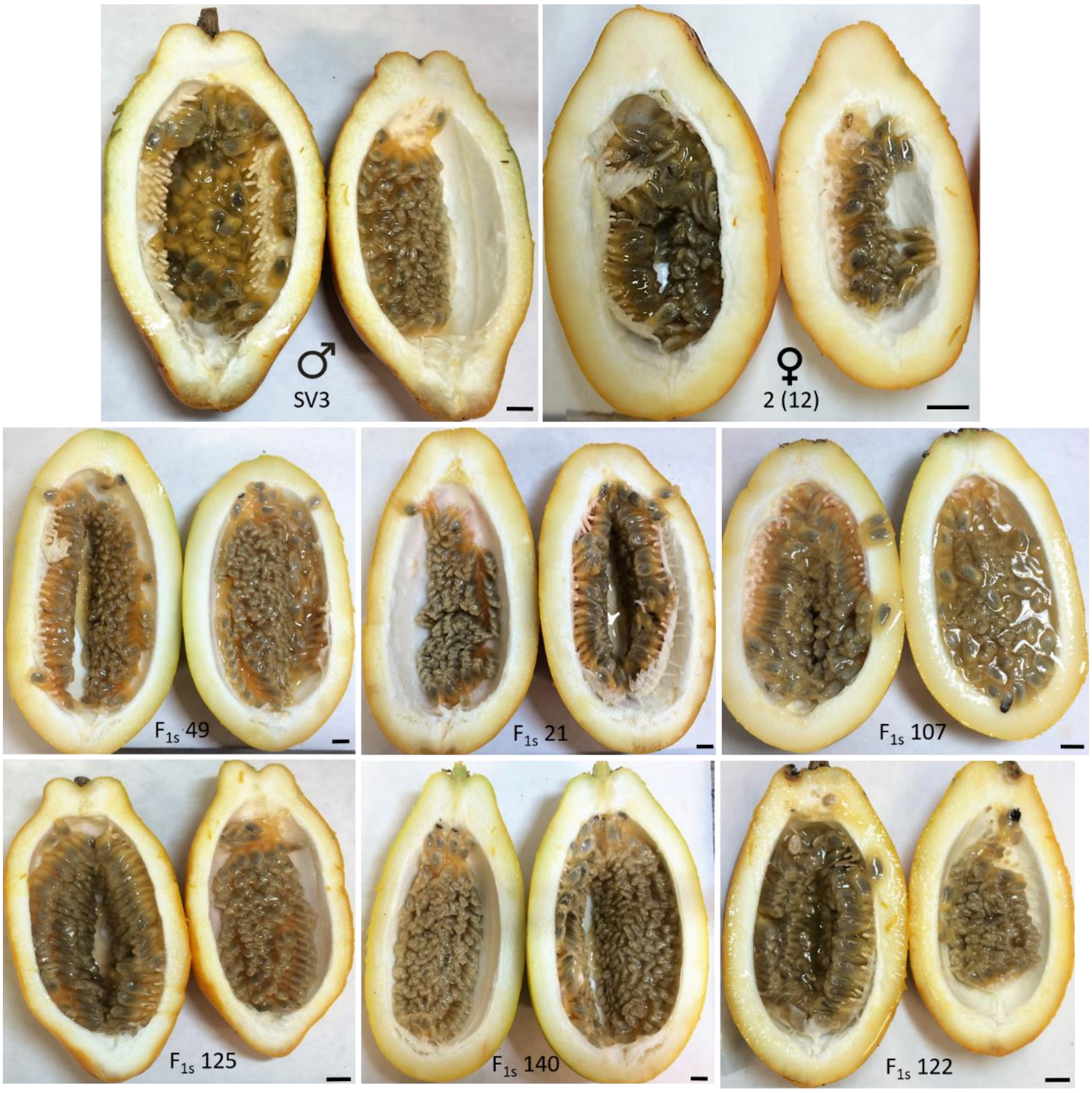

Supplementary material 4: Response to selection in sweet passion fruit. Note de difference in the thickness of skin and pulp content between fruits of the parental accessions (above). The six superior genotypes (below) were selected on the basis of a multiplicative index (MI). Bar = 1 cm.
