# Supplementary material for: Improving yield and fruit quality traits in sweet passion fruit: Evidence for genotype by environment interaction and selection of promising genotypes
Source: PLoS One. 2020 May 14;15(5):e0232818. doi: 10.1371/journal.pone.0232818 (PMC7224466; doi:10.1371/journal.pone.0232818)
Supplement: S2 Fig — (PDF) [file pone.0232818.s005.pdf]

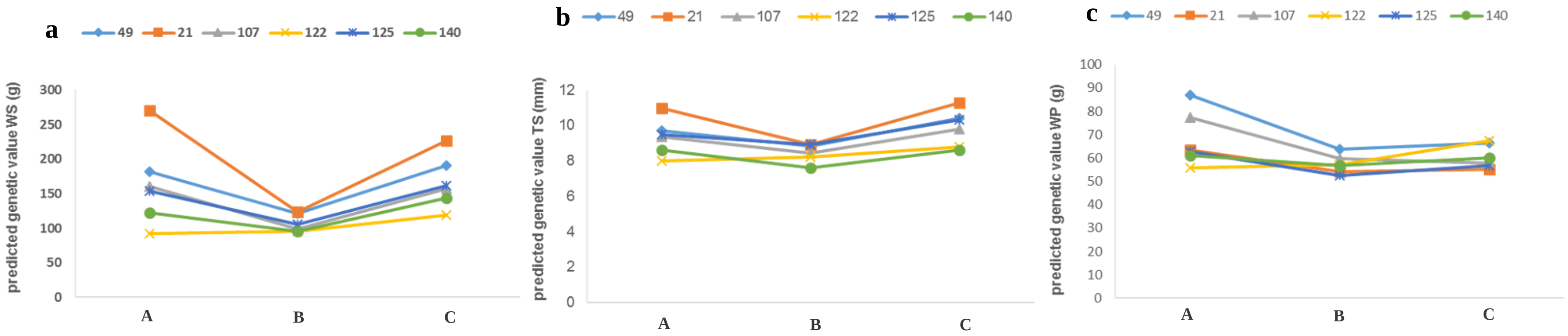

Supplementary material 5: Predicted genetic values of the six selected genotypes for Weight of Skin (a), Thickness of Skin (b) and Weight of Pulp (c) in each of the environments: A, B and C.
