# Supplementary material for: Improving yield and fruit quality traits in sweet passion fruit: Evidence for genotype by environment interaction and selection of promising genotypes
Source: PLoS One. 2020 May 14;15(5):e0232818. doi: 10.1371/journal.pone.0232818 (PMC7224466; doi:10.1371/journal.pone.0232818)
Supplement: Attachment — Submitted filename: Response to Reviewers_Chavarria et al.docx [file pone.0232818.s006.docx]

November 27, 2019

Manuscript ID PONE-D-19-24935

Ref. **Improving yield and fruit quality traits in sweet passion fruit: evidence for genotype by environment interaction and selection of promising genotypes**

*Lourdes Maria Chavarría-Perez^1^, Willian Giordani^1^, Kaio Olimpio das Graças Dias^1^, Zirlane Portugal da Costa^1^, Carolina Albuquerque Massena Ribeiro^1^, Anderson Roberto Benedetti^1^, Luiz Augusto Cauz-Santos^1^, Guilherme da Silva Pereira^2^, João Ricardo Bachega Feijó Rosa^1^, Antonio Augusto Franco Garcia^1^, Maria Lucia Carneiro Vieira^1*^*

Dear Professor David A. Lightfoot

Academic Editor

PLOS ONE

First, we would like to thank you and the reviewers for carefully reading our manuscript and for the constructive comments, which surely helped us to improve it. We have revised our paper in light of the reviewers' comments and made the necessary changes. A point-by-point response to these comments is provided below. We hope that the revised version is now suitable for publication in ‘PLoS ONE’.

Sincerely yours,

Maria Lucia Carneiro Vieira

Corresponding author

University of São Paulo, Brazil

**Reviewer 1**

**General comments**

*The authors have reported on “Improving yield and fruit quality traits in sweet passion fruit: evidence for genotype by environment interaction and cross-compatibility in selected genotypes.” The experimental design is generally adequate, and the flow of ideas is satisfactory. However, there is need a to succinctly define objective(s) of the study. Also, the manuscript seems to report on two separate studies which do not seem to converge into a coherent story. This situation masks the effectiveness of either. While the test for ‘genotype by environment interactions’ is fairly well developed, results reported and discussed, the less well developed ‘compatibility test’ takes up much of the conclusion.*

*The compatibility test results in some successful fruit formation. However, for the compatibility experiment to have been useful as part of this manuscript, the resulting progeny from these crosses could have been tested side by side with the other 30 ‘individuals’ in the different environments. As it is, the compatibility test is obviously a study of interest to breeders but is unfortunately out of place in this manuscript. I would suggest that it be developed as an independent study.*

*In all the section, a glaring deficiency is the lack of clarity and specificity of statements made; some of these require adequate citations. Besides statistical analyses and formulae terms should be adopted to the existing study experimental design.*

*The discussion section needs to be developed beyond simply reiterating what is reported in the results section. I am sure the quality of this manuscript will significantly improve if the following concerns are addressed and grammatical mistakes taken care of.*

**Response:** We deeply appreciate the reviewer’s comments which have surely contributed significantly to improving our manuscript. Regarding the objectives, we have reworded this section.

Apart from the main objective of our study, i.e. to obtain a representative picture of the 30 full sibs, we also aimed to identify promising material that could be used in future breeding programs (for instance, based on a recurrent selection approach) or even to make them available to producers. It is worth noting that the sweet passion fruit can be considered an orphan fruit species, especially in relation to the lack of improved varieties. Therefore it seemed essential to check whether the six selected genotypes would be used to generate breeding populations or in commercial orchards, which are fundamentally dependent on fertilization capacity. In some species of *Passiflora*, the promotion of outbreeding is attributed to a genetically controlled self-incompatibility (SI) system. The SI system in turn is controlled by the *S-*locus, comprising a few tightly linked genes coding for pollen–pistil recognition, which functions as a Mendelian multiallelic locus not influenced by external factors. In this context, we decided to perform the compatibility experiment in just one of the environments (C), during the 2017/18 and 2018/19 growing seasons.

**Abstract**

1. *Line 31: popularly not popular*

**Response:** We apologize for the mistake and have corrected the text: “…understudied crop, popularly known…”

2. *Line 32: “... is assumed to be a self-incompatible species ….” This portion of the sentence is unnecessary unless the ‘self-incompatibility’ is defined. I would leave it out. I believe it is still ok to include the sentence portion “…is highly appreciated for its typical aroma and flavor characteristics” without the first portion.*

**Response:** We accepted the reviewer’s suggestion and have removed the unnecessary portion of the sentence: “It is highly appreciated for its typical aroma and flavor characteristics.”

3. *Lines 33 -34: “With the aim of estimating the genetic and phenotypic parameters related to fruit traits…” Please be clear what genetic and phenotypic ‘parameters’ one should expect in the manuscript.*

**Response:** We thank the reviewer for this suggestion and have specified the parameters. The text has been modified as follows: “…with a view to estimate the heritability and genetic correlations, and investigating the GEI and response to selection for nine fruit traits (weight, diameter and length of fruit; thickness and weight of skin; weight and yield of fruit pulp; soluble solids, and yield). …”

4. *Lines 34 – 35: Please define the objective of this study succinctly. Hint: you can summarize what is in lines 104 – 107 (under ‘Introduction’).*

**Response:** We accepted the suggestion and have rewritten the objectives of our study under ‘Introduction’.

5. *Lines 36 - 37: ‘In this study, we reevaluated these superior genotypes in three environmental conditions. ----Q: Superiority based on what? Are these the 100 or the 30 genotypes? This is confusing if considered with: Lines 37 – 39: “The results of the multi-environment trial analysis indicate that the genotypes do not behave consistently across these environments, and this was taken into account when selecting genotypes.” …..Q: Were the trials conducted for the 100 or the 30 genotypes before ‘xy’ number of genotypes were selected? To remedy this confusion, I suggest lines 36 – 39 be rewritten…..**

**Response:** Please see the new text of the Abstract shown below. Also, the fragment “…The results of the multi-environment trial analysis indicate that the genotypes do not behave consistently across these environments, and this was taken into account when selecting genotypes…” has been excluded from the Abstract.

6. *Line 39 – 41: “Pairwise genetic correlations among the fruit traits were evaluated, and different genotype rankings obtained depending on the trait and environment, providing further evidence of genotype by environment interaction.” Up to this point, I do not know ‘the traits’ you are referring to. Please mention the traits in line 34.* Also, ranking based on…. what? Mention that basis here, instead of: ‘...depending on the trait and environment’. Please remove: ‘...providing further evidence of genotype by environment interaction’; there is no statement to support this in the abstract.

**Response:** We appreciate the suggestion. The traits have been referred to, the ranking specified and the fragment ‘… providing further evidence of genotype by environment interaction’ removed.

7. *Lines 41 – 42: “Finally, we used a multiplicative selection index to select 20% of genotypes…….”. Is this 20% of 30 genotypes mentioned in Line 35? Would that mean six (6) genotypes mentioned in line 217? Please be clear.*

**Response:** We have rewritten both fragments to make them clear. Please see the new text of the Abstract shown below.

8. *Lines 44 – 47: “The consensus is that open-pollinated populations can be used as commercial varieties in crop species that are sensitive to inbreeding depression or within breeding programs that are not well developed. For these reasons...” ---------what purpose does this statement serve considering the study objective (which you have not defined succinctly)? Please remove it; otherwise it belongs in other sections e.g., introduction or discussion sections.*

**Response:** Please see the new text of the Abstract shown below.

The new **Abstract** reads as follows:

Breeding for yield and fruit quality traits in passion fruits is complex due to the polygenic nature of these traits and the existence of genetic correlations among them. Therefore, studies focused on crop management practices and breeding using modern quantitative genetic approaches are still needed, especially for *Passiflora alata*, an understudied crop, popularly known as the sweet passion fruit. It is highly appreciated for its typical aroma and flavor characteristics. In this study, we aimed to reevaluate 30 genotypes previously selected for fruit quality from a 100 full-sib sweet passion fruit progeny in three environments, with a view to estimating the heritability and genetic correlations, and investigating the GEI and response to selection for nine fruit traits (weight, diameter and length of the fruit; thickness and weight of skin; weight and yield of fruit pulp; soluble solids, and yield). Pairwise genetic correlations among the fruit traits showed mostly intermediate to high values, especially those associated with fruit size and shape. Different genotype rankings were obtained regarding the predicted genetic values of weight of skin, thickness of skin and weight of pulp in each environment. Finally, we used a multiplicative selection index to select simultaneously for weight of pulp and against fruit skin thickness and weight. The response to selection was positive for all traits except soluble solids, and the 20% superior (six) genotypes were ranked. Based on the assumption that incompatibility mechanisms exist in *P. alata*, the selected genotypes were intercrossed in a complete diallel mating scheme. It is worth noting that all genotypes produced fruits, which is essential to guarantee yields in commercial orchards.

**Introduction**

9. *Lines 107 to 112: I suggest you remove these sentences. They are unnecessary here.*

**Response:** We acknowledge the suggestion and have excluded these sentences.

10. **Line 109 – 112: Was this one of the objectives of the study, or was it just part of a necessary procedure to enable the evaluation of the actual objective? If so, I suggest you restrict it to the materials and methods. Otherwise, remove it.*

**Response:** In fact, due to the positive results we have obtained, it has become an objective of our study to identify and rank the superior genotypes applying 20% intensity of selection.

Therefore, to make this point clear to readers, the last sentence of the Introduction section has been changed as follows:

“As a consequence of our results, we have identified six superior genotypes, which were subsequently intercrossed in all combinations, including the reciprocals. Based on the assumption that incompatibility mechanisms exist in *P. alata* [13], the fruit set capacity in all pollinations has also become a goal of our study”.

**Materials and Methods**

11. *General: Please number the equations so it is easy to reference them when needed.*

**Response:** We appreciate the suggestion and have numbered all the equations.

12. *Please start by rewriting the statement in lines 124 – 127, thus: In this study, we examined 32 genotypes, consisting of a sample (n = 30) of full-sib progeny of sweet passion fruit and the two parents. The two parents are outbred and divergent accessions. Then continue from line 116: “The male parent……………”*

**Response:** The statement has been rewritten as recommended:

“In this study, we examined 32 genotypes, consisting of a sample (n = 30) of full-sib progeny of sweet passion fruit and the two parents. Both parents are outbred and divergent accessions. The male parent, denoted SV3, was an indoor…”

13. *Line 115: “Instead of ‘population (N= 30)’, use: sample (n = 30)*

**Response:** All “Ns” have been replaced with “n”.

14. *Line 124: Describe the progeny, e.g., The 30 full-sibs were part of a progeny of 100 individuals that had been evaluated previously in two ….continue with line 127. Also see comments elsewhere in the discussions.*

**Response:** The sentence has been changed exactly as recommended.”

15. *Line 129: replace “three environments” with: two locations and two seasons, consolidated for a total of three environments (A, B and C) for the purpose of this study. Environment A and B were represented by seasons …………… While Environment C was represented by 2nd season (dates) at location…..…….*

**Response:** We acknowledge the reviewer’s suggestion. The sentence has been changed exactly as recommended.

16. *Lines 130 – 133: Please name the locations in addition to the grid references.*

**Response:** We have added the names of the locations as recommended.

17. *Lines 143 – 146: Please include the manufacture’s name in addition to instrument specification.*

**Response:** The manufacture’s name has been included.

18. *Lines 157 – 167: In the first two model equations, a ‘blocking’ variable has been used, while in the experimental design, it is not explicit what the blocks are. Advice: please adapt the equations to the experimental design; what you have here looks a lot more generic. Either the blocks will need to be defined considering the existing experimental design, or you need to modify the equations to suit your design. This is important.*

**Response:** We thank the reviewer for this comment and have added a brief description of the blocks. “…with the blocks arranged according to the field slope with three plants per plot arranged in rows.”

19. *Line 180 – 181: Briefly explain the importance (justification for) of using AIC and BIC for VCOV structure variables in your LMM analysis. A single sentence will do.*

**Response:** We have explained the importance of AIC and BIC as follows: “These criteria allow comparison of models with different random terms or VCOV structures, with a common fixed part, and evaluation the goodness-of-fit of each model, even when they are not nested.”

20. *Line 196: Please use small ‘s’ not ‘sigma’ in the equation for CV for your sample distribution.*

**Response:** We acknowledge the reviewer’s comment. However, most of the statistical geneticists who have been applying mixed models to genetic data also use ‘sigma’ to refer to genetic variances. For this reason, we prefer to keep ‘sigma’. Please see Lu et al. (2017) and Li et al. (2018).

21. *Line 197: “…the phenotypic average for the sample” instead of “the average for the population”. Remember you are working with sample means. Fortunately, in this particular case, the CV values are not going to change.*

**Response:** We thank the reviewer for the suggestion and have rewritten the fragment as: “…is the phenotypic average for the sample and…”

22. *Line 215: “…incompatibility”. Please define this term. Did you mean reproductive incompatibility?*

**Response:** We apologize for using this term. In fact, we intended to refer to the self-incompatibility system, a widespread outcrossing system present in roughly half of the flowering plants, including cultivated species. Many hermaphroditic plants utilize this system as a mechanism to maintain [genetic diversity](https://www.sciencedirect.com/topics/biochemistry-genetics-and-molecular-biology/genetic-divergence) within a population by rejecting self- or genetically-related pollen at the stigma surface. This seems to be the case for *P. alata* (please see Ocampo et al., 2016).

Thus, the text has been modified as follows:

“Due to the potential for reproductive self-incompatibility in *P. alata*, which does not allow self-fertilization or fertilization involving genetically related individuals, the selected genotypes were crossed in a 6 × 6 diallel design, and the fruit set capacity evaluated at the same site (C), during the 2017/18 and 2018/19 growing seasons”.

23. *Line 216 - 218: “As potential incompatible crosses might occur due to the existence of incompatibility mechanisms in P. alata, the selected genotypes were crossed and fruit set evaluated at the same site (C), using a complete 6 × 6 diallel cross during the 2017/18 and 2018/19 growing seasons” ------Please consider rewriting this sentence. Example: Due to the potential for reproductive self-incompatibility in P. alata, the selected genotypes were crossed in a 6 × 6 diallel design, and fruit set evaluated at the same site (C), during the 2017/18 and 2018/19 growing seasons.*

**Response:** Please see the response to Q22.

24. *Line 219: Remove ‘artificially’.*

**Response:** We have replaced “artificially” with “manually”

25. *Line 225 – 226: What are “10 pollinations”? Instead, write how many (female) plants were pollinated.*

**Response:** We agree and have rewritten the fragment as: “At least 10 stigmas were hand-pollinated for each cross…”.

26. *Line 228 – 229: Were there some crosses producing 0 < fruit set < 50? How were these classified, or were they simply ignored (assume the reader has not looked at results - Table 4)*

**Response:** We have included a sentence elucidating this issue: “The crosses were considered compatible (+) when > 50% of the pollinated flowers set fruits (Figure 1e-g) and incompatible (–) in the absence of fruit set (Figure 1h-j). Otherwise they were considered partially compatible.”

**Results**

27. *Lines 236 – 250: See comments for lines 180 – 181.*

**Response:** In the Materials and methods section we have added a justification for using AIC and BIC. Here, in the Results section, we have explained the criteria for selecting the “…most appropriate model for each trait (Table 1), given that lower AIC or BIC values indicate a better fit.”

28. *Line 252 with reference to Table 2: what is the difference between WP and YP (how do you determine WP as opposed to YP)? Is the DF longitudinal or lateral? Please add this information in the materials and method under ‘….Measurements’ (Line 128).*

**Response:** We have added an explanation of how we computed YP: “… (YP, estimated as the quotient between WP and WF) …”. Also, the information on DF has been included under “Field Sites and Measurements”: …“diameter at the widest lateral point”.

29. *Lines 253 – 256: “Furthermore, the random effect of GEI significantly affected all traits. These findings also indicate that genotypes do not show consistent behavior across environments, and this should be taken into account when selecting genotypes.” -----These inferences cannot be made from Table 2 as it is. These is need for a summarized effects table showing whether G, GxE, e (e for residual) are significant for each trait. Remember you used REML to model the ‘1st’ and ‘2nd’ equations. I need to see some threshold to declare significance. This should be fairly simple; the good thing, you already have data!*

**Response:** We thank the reviewer for this comment and have cited S1 Table that refers to the significant effect of GEI.

30. *Line 266 - 271: What is a “low values of CV” that defines “good experimental precision” ? Please explain briefly and provide some reference (citation). Also, please compare the same trait in the three environments, not between one trait in one environment and another trait in a different environment.*

**Response:** We have included a citation and an example of CV values for the DF trait (diameter at the widest lateral point of the fruit) in the three environments:

“…According to [23], CVs are expected to range between 5 and 15% in field experiments. The predominantly low CVs (<10%) obtained indicate good experimental precision. The only exception was Yield, in which high CV values were estimated regardless of the environment. Furthermore, the estimated CVs for each trait in A, B and C were very similar, denoting that the experimental accuracy was comparable among environments. For instance, the CV values for DF were the lowest in all three environments (6.59% in A; 6.11% in B and 5.48% in C).”

31. *Line 271: “…denoting that all experimental conditions were equally reliable.”----- Equally variable? How was this tested? CV for Yield is 71.68 in Environment A, but it is 52.07 in Environment C. Also, the means are 1.90 in A, but 3.98 in C. The ranges look a lot closer between A and C, but not between these two environments and B. What does this imply?*

**Response:** According to Snedecor and Cochran (1980), due to the dimensionless nature of the coefficient of variation, it allows researchers to compare the accuracy of experiments despite the different units of measurement, and has been applied to compare results from different experiments and conditions (please see Steel et al., 1997). As mentioned by the reviewer, except for Yield, low CV values were estimated regardless of the environment and trait, indicating good experimental precision. Nevertheless, we agree with the reviewer that asserting “all experimental conditions were equally reliable” is too strong an inference, requiring a formal test. For this reason we have reworded the text as follows: “… denoting that the experimental accuracies were comparable among environments.”

With regard to similarity among environments, as shown by the genetic correlation between environments for all traits (S2 Table), between A and B, *r* _A, B_ ranged from 0.17 to 0.95 (DF and YP, respectively), whereas between A and C, *r* _A, C_ ranged from 0.39 to 0.96 (WP and SS, respectively) implying a higher correlation between A and C than between these two environments and B.

Finally, as mentioned in the text: “…This pattern of correlation values has implications for genotype ranking, depending on the trait and environment, lending further weight to the existence of GEI.”

32. *Lines 273 – 283 with reference to Figure 2: The fit lines for the correlation plots for LF-SS; TS-SS, and WS-SS appear positive, while the correlation plot for YP-SS appears to be negative. Are the nature of the corresponding correlation values correct? Also see lines 321 – 323.*

**Response:** We have double-checked all the correlation values and they are correct. We believe that the reason for this apparent conflict is that the red lines in the scatterplots are not linear regressions, but locally smoothed regressions.

33. *Line 312 … ‘accounted for’, not ‘account for’*

**Response:** We apologize for this error and have corrected it.

34. *Lines 337 – 339: ‘The AEC abscissa is the straight line that passes through the biplot origin and the “average environment”, and the AEC ordinate is the line perpendicular to the abscissa. The projections of the genotype on the abscissa represent the main genetic effects and therefore rank the genotypes in relation to their mean performance’. ------This extra information is not necessary here. Please reference the relevant biplot figure and let the reader glean this information in the corresponding caption for that figure.*

**Response:** We thank the reviewer for the suggestion and have removed the unnecessary information. However, the second statement was retained as a link with the next sentence, and because some readers might not be familiar with GG biplot analysis and the projections represent the main genetic effects, allowing genotype ranking.

“The projections of the genotype on the abscissa represent the main genetic effects and therefore rank the genotypes in relation to their mean performance. Thus, in accordance with this ranking, the genotypes were classified according to yield, as follows: …”

35. *Lines 342 – 345: Which figure is being referenced here? Figures 5a and 5b? Some numbers are hardly legible.*

**Response:** We have now referenced Figure 5b in the text.

36. *Lines 378 – 394: It is my opinion that if this section is not a procedure that enables the achievement of the main objective, it does not belong in this manuscript. Thirty lines have been tested in different environments for variations in traits associated with fruit anatomy and quality. The germplasm have been ranked using the procedures described in the text. It is not clear how testing reproductive compatibility (self and outcrosses) and the results of such tests have added value or relevance to the tenor of this manuscript. It is confusing at best.*

**Response:** We agree with the reviewer that the reproductive compatibility test adds distinct results compared to the GEI studies. However, there is a lack of improved varieties of sweet passion fruit. In this scenario, it seems imperative to check whether the six selected genotypes can be used in commercial orchards, which is heavily dependent on their fertilization capacity. In addition, these genotypes are promising candidates for further breeding studies. This knowledge will have positive impacts, introducing new perspectives for producers and researchers interested in this tropical fruit.

37. *Figure 2: The direction of the fit line seem to conflict with the sign assigned to the corresponding r: LF:SS, TS:SS, WS:SS, line shows positive orientation, but the corresponding r values are negative. YP:SS, line appears positive, yet r is negative (?!). Please check to see this is correct.*

**Response:** We have double checked all the correlations and they are correct. We believe that the reason for this apparent conflict is that the red lines in the scatterplots are not linear regressions, but locally smoothed regressions.

**Discussion**

38. *Line 407 - 411: N=180 is being introduced here for the first time? I want to see this description in the materials and method, under plant materials; it tells us how the material in the present study was arrived at.*

**Response:** This extra information is not necessary in the paper; in fact this is the number (n = 180) of individuals used to construct a linkage map for the same population and already published (Pereira et al., 2013).

39. *Line 411: was sampling random or not for the 100 ‘individuals’?*

**Response:** In fact, the selection of the 30 individuals was performed by means of a multiplicative selection index:

“…One hundred individuals were then sampled and field-evaluated, and QTL mapping analysis performed to identify loci associated with fruit quality traits. The MSI was also used to select the 30 most promising genotypes [6]...”

40. *Line 414: ….reevaluated these full-sibs. Add ‘30’ before ‘full-sibs’.*
41. *Line 415: replace ‘to confirm that’ with ‘to determine if’.*

**Response:** We accepted both the reviewer’s suggestions and have made the respective changes.

42. *Lines 425 – 426: Add citation at the end of this sentence.*

**Response:** We have added the two citations: “…sets of genotypes are evaluated in different trials [30,31].”

43. *Line 434: As stated in an earlier comment, what do you consider as low CV? I suggest you also incorporate what the CVs tell about the variation of the traits between the environments.*

**Response:** Please see the response given to Q30.

44. *Lines 438: which _based on. Add ‘was’*

**Response:** We apologize for the mistake and have corrected the text: “…Yield, which was based…”.

45. *Lines 442 – 444: “In the original population (N= 100), high heritabilities for the same fruit traits (except Yield) were estimated, varying from 0.59 (WP) to 0.82 (SS).”------Are these results shown anywhere in the manuscript, or published elsewhere? And what is ‘original’ population?! N=180, 100, 30 or 6? I suggest you purge the term ‘original’ here and elsewhere with reference to population. Also, remember you are working with a small sample of 30.*

**Response:** The results are shown in Pereira et al. (2017). To avoid any misunderstanding, we have excluded the term “original” elsewhere with reference to n = 100, and have cited Pereira et al. (2017) instead.

46. *Lines 444 – 446: any citations?*

**Response:** We have now added a citation.

47. *Lines 457 – 461: see comments for lines 442 – 444, and elsewhere.*

**Response:** Please see the response to Q45.

48. *Line 463: What are ‘dissociations’ in this context? Define it or replace with a more appropriate word.*

**Response:** We thank the reviewer for this suggestion and have replaced ‘dissociations’ with a more appropriate term: “… reductions in the magnitudes of correlations...”

49. *Lines 470 – 473: What does the genetic correlation between traits tell you about the variability in the phenotypes? How is this important to breeders? Please add a brief discussion on this.*

**Response:** We have included the following statement:

“ …What is particularly interesting is that these reductions in the magnitudes of correlations were obtained with only one cycle. If there are strong correlations, selection based on a single trait might result in an increment of undesirable traits, as occurred for WS and TS when individuals were selected for WP. For breeders, even a slight detachment of correlated traits is of great interest, since it allows selection for one trait with little impact on the others, denoting that using a selection index might be appropriate when highly correlated traits are targeted in breeding programs.”

50. *Line 505: As posted earlier, what is the substantive difference between WP and YP?!*

**Response:** WP refers just to the weight of the pulp, obtained using a digital scale; YP is calculated as the quotient between WP and WF. Therefore YP is a measurement that tells us how much of the fruit mass might be attributed to its pulp.

51. *Lines 518 – 537: Decide if this study was a reproductive compatibility test or not. Otherwise, I do not see the need for these conclusions here. See comments elsewhere.*

**Response:** We agree that the previous text was not clear on this point. Please see the responses given above and the following paragraph added to the Conclusion section.

“In conclusion, this study shows that many of the phenotypic differences are due to genetic variation, allowing high heritability estimates. Although strong genetic correlations were detected for most traits, we were able to demonstrate that the use of a selection index could help reduce the magnitudes of correlations between desirable and undesirable traits. This index allowed the selection of six promising genotypes that are also mostly cross compatible and therefore can be used commercially or for continuing the breeding scheme. Finally, our results provide a comprehensible view of the genotype by environment interaction and allowed us to interpret how the sweet passion fruit genotype performs across environments.”

**Reviewer 2**

**General comments**

*The manuscript presented the phenotypic evaluation of 30 full-sibs in a different environment. The objective is clear, and the methods and results are well presented. Statistical models were used to comprehend the potential and performance of each line and selections were made as reported in the manuscript. However, a whole set of the populations should have been used in the evaluation. The genotype data that have been already generated (Pereira et al., 2017) could have been used to conduct a detailed mapping and genetic characterization of the trait, in addition to genotype by environment interaction studies. Such effort would make the manuscript much stronger and of interest to wider readers of the journal.*

**Response:** We strongly agree with the reviewer regarding the importance of conducting detailed QTL mapping to characterize the traits. In fact, this analysis has already been performed using the same population (n = 100 individuals) by Pereira et al. (2017), who aimed to characterize the genetic architecture of QTLs underlying fruit quality traits. Here we used 30 genotypes selected from the former population and reevaluated them in three environments in order to achieve two main objectives: i) to estimate the heritability coefficient and genetic correlations, and investigate the genotype by environment interaction in order to predict genetic values for nine fruit traits; and ii) to produce a more representative picture of the 30 selected genotypes.

In view of the importance of accurately interpreting the GEI and efficiently selecting superior genotypes, we focused on the previously selected individuals and reevaluated them in three environments with a high number of replicates. A randomized block design with six (environment A) and three (environments B and C) replicates was used to cover wide experimental areas.

The size of the population that we evaluated here (n = 30) is not sufficient to perform the kind of linkage analysis carried out by Pereira et al. (2017). Finally, we do believe that the present study represents an important contribution for investigators interested in tropical plant species. For instance, the existence of a self-incompatibility mechanism was experimentally demonstrated in a full sib population of *P. alata*. Importantly, we showed that many of the phenotypic differences are due to genetic variation, allowing high heritability estimates. Although strong genetic correlations were detected for most traits, we were able to demonstrate that the use of a selection index might help reduce the magnitudes of correlations between desirable and undesirable traits. This index allowed the selection of six promising genotypes that are also mostly cross compatible and therefore can be used commercially or for continuing the breeding scheme. Finally, our results provide a comprehensible view of the genotype by environment interaction and allowed us to interpret how the sweet passion fruit genotype performs across environments.

**References**

Bernardo, R. (2010). *Breeding for quantitative traits in plants*. 2nd ed. Stemma Press: Woodbury, MN.

Dawson, J. C., Endelman, J. B., Heslot, N., Crossa, J., Poland, J., Dreisigacker, S., Manes, Y., Sorrells, M. E., Jannink, J. L. (2013). The use of unbalanced historical data for genomic selection in an international wheat breeding program. *Field Crops Research*, 154, 12-22.

Holland, J. B., Nyquist, W. E., & Cervantes-Martínez, C. T. (2010). Estimating and Interpreting Heritability for Plant Breeding: An Update. *Plant Breeding Reviews*, 9–112. doi:10.1002/9780470650202.ch2

Li, Y., Dungey H. S., Carson, M., Carson S. (2018) Genotype by environment interaction for growth and *Dothistroma* resistance and clonal connectivity between environments in radiata pine in New Zealand and Australia. *PLOS ONE* 13(10): e0205402. https://doi.org/10.1371/journal.pone.0205402

Lu, X., Luan S., Cao B., Meng X., Sui J., et al. (2017) Estimation of genetic parameters and genotype-by-environment interactions related to acute ammonia stress in Pacific white shrimp (*Litopenaeus vannamei*) juveniles at two different salinity levels. *PLOS ONE* 12(3): e0173835. https://doi.org/10.1371/journal.pone.0173835

Ocampo, J., Arias, J. C. & Urrea, R. Interspecific hybridization between cultivated and wild species of genus Passiflora L. *Euphytica* (2016) 209: 395. https://doi.org/10.1007/s10681-016-1647-9

Pereira, G. S., Nunes, E. S., Laperuta, L. D. C., Braga, M. F., Penha, H. a., Diniz, a. L., et al. (2013). Molecular polymorphism and linkage analysis in sweet passion fruit, an outcrossing species. *Annals of Applied Biology* 162, 347–361. doi:10.1111/aab.12028.

Pereira, G. S. da S., Laperuta, L. D. C., Nunes, E. S. E. S., Chavarría-Perez, L., Pastina, M. M. M. M., Gazaffi, R., et al. (2017). The sweet passion fruit (*Passiflora alata*) crop: genetic and phenotypic parameter estimates and QTL mapping for fruit traits. *Tropical Plant Biology* 10, 18–29. doi:10.1007/s12042-016-9181-4.

Snedecor, G. W., Cochran, W. G. (1980) Statistical methods. 7ed. Ames: The Iowa States University Press. 593p.

Steel, R. G. D., Torrie, J. H. & Dickey, D. A. 1997. *Principles and procedures of statistics, a biometrical approach*. 3rd ed. McGrawHill Book Co., New York, NY.
