# Supplementary material for: Improving yield and fruit quality traits in sweet passion fruit: Evidence for genotype by environment interaction and selection of promising genotypes
Source: PLoS One. 2020 May 14;15(5):e0232818. doi: 10.1371/journal.pone.0232818 (PMC7224466; doi:10.1371/journal.pone.0232818)
Supplement: Attachment — Submitted filename: Response to Reviewers_PLoS ONE_Teodoro.docx [file pone.0232818.s007.docx]

Manuscript ID PONE-D-19-24935

Ref. **Improving yield and fruit quality traits in sweet passion fruit: evidence for genotype by environment interaction and selection of promising genotypes**

**Additional Editor Comments (if provided):**

*Dear authors, the manuscript PONE-D-19-24935R1 is the result of great work and has merit for publication. However, there are several inconsistencies in the statistical procedures that must be corrected before I consider it for publication. Another important point, English has several technical errors and needs to be reviewed by a specialized company. For this reason, I invite the authors to respond point-by-point to my comments below and that of Reviewer 3 in a reply letter. Check all changes made to the text in red. It is not necessary to use change tracking.*

***1.*** *The authors used Linear mixed-model analysis using the ASReml-R package for individual and joint analyzes of variance. Therefore, it is necessary to write equations 1 and 2 in the form of matrix notation;*

**Answer:**

As suggested, equations 1 and 2 are now written in matrix notation (please see p. 7, in red).

***2.*** *The authors used an identity matrix in these analyzes (matrix I). However, it is possible to estimate the kinship matrix for the assessed population;*

**Answer:**

We thank the editor for his pertinent comment. The reason for not modeling the genotypic effects by including the kinship matrix is based on conceptual and technical aspects.

First, in conceptual terms, if we had added the information on the kinship matrix for the assessed population, the breeding values we would have obtained would reflect only the additive effects. As the species we are working on (*Passiflora alata*) allows vegetative propagation (cloning), we can exploit more than just the additive effects.

Regarding the technical issues, as all the genotypes evaluated in our study are full-siblings, their expected coefficients of coancestry are the same, making the off-diagonal of the kinship matrix the same for all of them. In this case the kinship matrix is expected to exploit the same amount of information from all of the genotypes (Bernardo, 2010), with no additional gains for our analyses.

Hence, although we are aware that kinship information is very useful if the aim is to predict breeding values, based on our breeding objectives and the structure of our population, an identity matrix serves our purposes.

***3.*** *Move Table 1 for supplementary material. In its place, provide a new Table containing the results of the LRT test for each trace in each environment and for each trace considering the joint analysis;*

**Answer:**

We have addressed this comment by moving Table 1 to Supplementary material. Results of the LRT test for the single analysis (previously in Supplementary material 1) are now presented in Table 1. Furthermore, we have performed the LRT test for the multi-environment trials, as suggested, and included the results in the same Table (see p. 12).

***4.*** *Include in Table 2 the genetic parameters for each trait considering the joint analysis;*

**Answer:**

We thank the editor for this comment and have added the genetic parameters estimated by means of joint analysis to Table 2 (see p. 13-14).

***5.*** *Lines 203 and 204: "genotypic correlations among traits were calculated for adjusted means, such as the Pearson coefficient, using program R". What did the authors mean by that? How were genotypic correlations estimated? Were they estimated from the BLUPs obtained? Further detail this procedure.*

**Answer:**

Genotypic correlations were estimated from the BLUPs, which were obtained from the best model selected based on AIC and BIC for the different tested VCOV (variance and covariance) structures. To make it clear we have included a sentence elucidating this issue (please see p. 8).

**6.** *The authors used the GGE biplot method to investigate the patterns of the GEI interaction. Although I really like this method and use it in several of my papers, it is not suitable for this case. The GGE biplot method is indicated for a large number of environments, which is not the case in this study. In the case of a few environments (n = 3) in this manuscript, I suggest the authors use the HMPRGV of Resende (2007) method. As the authors are working with mixed models and have unbalances, this method would be ideal to demonstrate the genotypes that have greater adaptability and stability.*

**Answer:**

We appreciate the editor’s suggestion. The HMPRGV was estimated for each trait and is presented below.

**Table. Harmonic Mean of Relative Performance of Genotypic Values (HMRPGV) for nine fruit traits, assessed in a set of 30 genotypes (full-sibs) of sweet passion fruit across three environments.**

| **Genotype** | **WF** | **DF** | **LF** | **TS** | **WS** | **WP** | **SS** | **YP** | **Yield** |
| --- | --- | --- | --- | --- | --- | --- | --- | --- | --- |
| 20 | 171.38 | 70.94 | 109.04 | 8.58 | 120.03 | 49.46 | 15.51 | 28.93 | 1.71 |
| 21 | 232.50 | 77.97 | 125.24 | 10.39 | 172.21 | 48.30 | 15.46 | 19.96 | 1.56 |
| 44 | 126.17 | 64.53 | 104.66 | 7.92 | 86.70 | 33.66 | 15.43 | 30.27 | 1.35 |
| 48 | 150.13 | 66.85 | 107.65 | 10.06 | 110.74 | 36.73 | 17.23 | 24.63 | 1.11 |
| 49 | 219.26 | 75.41 | 123.46 | 9.77 | 154.35 | 58.08 | 15.74 | 27.18 | 1.50 |
| 52 | 120.57 | 62.45 | 108.28 | 7.81 | 77.13 | 42.91 | 16.46 | 31.92 | 0.41 |
| 68 | 169.88 | 70.21 | 116.90 | 9.42 | 121.98 | 43.94 | 16.25 | 25.82 | 1.41 |
| 69 | 112.39 | 63.23 | 109.07 | 7.72 | 74.21 | 38.75 | 18.45 | 28.36 | 0.20 |
| 76 | 166.98 | 69.98 | 119.58 | 9.31 | 114.85 | 43.28 | 16.08 | 26.83 | 0.83 |
| 83 | 199.46 | 71.65 | 121.74 | 10.21 | 153.53 | 39.14 | 15.64 | 19.74 | 1.02 |
| 85 | 186.48 | 71.32 | 113.39 | 10.37 | 140.55 | 40.42 | 14.73 | 22.72 | 1.63 |
| 87 | 143.69 | 65.54 | 109.35 | 8.67 | 101.04 | 41.92 | 16.15 | 27.77 | 0.35 |
| 91 | 152.35 | 68.46 | 102.10 | 9.16 | 107.32 | 42.77 | 15.85 | 28.31 | 0.84 |
| 92 | 152.63 | 67.02 | 113.38 | 8.80 | 103.28 | 44.67 | 17.04 | 30.45 | 1.05 |
| 93 | 117.29 | 64.05 | 111.89 | 5.62 | 75.15 | 40.65 | 16.65 | 27.64 | 0.44 |
| 100 | 158.30 | 69.29 | 112.07 | 9.02 | 109.39 | 43.20 | 15.59 | 27.80 | 0.92 |
| 107 | 188.47 | 71.15 | 117.05 | 9.25 | 126.16 | 51.25 | 15.80 | 27.27 | 1.42 |
| 110 | 160.42 | 70.16 | 112.94 | 8.37 | 109.94 | 45.92 | 16.80 | 26.19 | 1.40 |
| 115 | 147.77 | 67.06 | 114.34 | 8.38 | 110.27 | 19.90 | 16.00 | 14.03 | 1.01 |
| 122 | 162.88 | 68.43 | 112.42 | 8.41 | 102.31 | 57.85 | 16.94 | 39.54 | 1.50 |
| 125 | 185.63 | 73.03 | 109.54 | 9.65 | 129.41 | 51.12 | 16.17 | 27.02 | 1.25 |
| 136 | 165.05 | 69.40 | 112.55 | 9.36 | 119.99 | 39.26 | 15.38 | 25.07 | 1.47 |
| 138 | 177.23 | 71.22 | 109.63 | 10.17 | 126.80 | 43.26 | 16.98 | 25.80 | 1.50 |
| 140 | 174.53 | 73.04 | 114.11 | 8.24 | 113.56 | 53.26 | 15.23 | 30.56 | 1.64 |
| 144 | 168.15 | 70.69 | 124.96 | 8.73 | 129.32 | 34.86 | 15.73 | 20.60 | 1.28 |
| 145 | 175.99 | 72.62 | 124.07 | 9.02 | 127.24 | 45.29 | 16.76 | 27.14 | 1.55 |
| 150 | 168.90 | 66.14 | 115.65 | 9.14 | 120.25 | 44.95 | 15.36 | 26.19 | 1.84 |
| 151 | 143.43 | 67.81 | 105.25 | 8.31 | 100.65 | 36.58 | 15.92 | 27.76 | 1.34 |
| 152 | 165.52 | 69.58 | 114.51 | 8.29 | 119.01 | 43.46 | 16.07 | 23.47 | 1.73 |
| 154 | 150.75 | 68.07 | 111.01 | 8.30 | 101.14 | 43.63 | 16.53 | 31.48 | 1.22 |
| 2(12) | 136.57 | 66.43 | 105.39 | 9.17 | 102.70 | 33.99 | 17.78 | 23.32 | 0.52 |
| SV3 | 142.73 | 68.18 | 108.98 | 7.95 | 98.25 | 38.84 | 14.92 | 29.77 | 1.59 |

WF = weight of fruit; DF = diameter of fruit; LF = length of fruit; TS = thickness of skin; WS = weight of skin; WP = weight of pulp; YP = yield of pulp; SS = total soluble solids.

Please note that there are no relevant differences in the results produced by HMPRGV compared to those produced by the GGE Biplot method. Furthermore, even though there are small differences, the general conclusions drawn from both analyses are the same. For example, in our manuscript we showed that “…genotype 21 is positioned at the polygon vertex indicating high performance for these traits (WF, LF, DF, WS and TS)”. The table above shows that this behavior is consistent with results obtained by the HMPRGV method. Also corroborated with HMPRGV, we report that “…other interesting genotypes are 49, 107 and 140, which appear in the sector formed by WP and Yield, and genotype 122 that also showed greater YP.” This coincidence between the GGE Biplot and HMPRGV results has also been reported in studies comparing these approaches in large (n ≥ 8 Santos et al. 2016) and small numbers of environments (n ≤ 4; Oliveira et al. 2018; Abreu et al. 2019; Santos et al. 2019).

Concerning the editor’s comment regarding the limited number of environments, it is important to note that in our study GGE Biplot analysis was fitted by trait-environment combination. Thus, considering that we have three environments and nine traits, our *n* is equal to 27 (Figure 4). Since the results are similar in both cases and with a view to exploiting the graph interpretation allowed by the GGE Biplot, we prefer to keep this analysis in our manuscript.

**7.** *In the face of such corrections, the authors will need to modify the end of the Introduction. In addition, the Results will be substantially modified and, consequently, the Discussion.*

**Answer:**

We have carefully addressed all these comments. Changes in the manuscript text are highlighted in red (see p. 7-8, 11-15, 23 and 25).

**Reviewers' comments:**

***Reviewer #3:*** *The manuscript shows important and novelty information about phenotypic evaluation of 30 sweet passion fruit full-sibs in three a different environment, and contributes with relevant results for the scientific community and researchers interested in this study. the objective of this study was reevaluate 30 genotypes previously selected for fruit quality from a 100 full-sib sweet passion fruit progeny in three environments, with a view to estimate the heritability and genetic correlations, and investigating the GEI and response to selection for nine fruit traits (weight, diameter and length of the fruit; thickness and weight of skin; weight and yield of fruit pulp; soluble solids, and yield). The paper is acceptable in terms of methods and procedures.*

**Minor corrections**

1. *Line 45, 106, 120, 154, 222, and 253 - Indent the first line of a paragraph*.
2. *Page 16 Line 37, Page 17 line 81, page 20 line 149, page 21 line 183, page 22 line 207 - Indent the first line of a paragraph*.

**Answer for 1 and 2:**

We thank the reviewer for the suggestion and have added indents to all the first lines of paragraphs.

3. *Page 15 line 28: What is reason for such a high coefficient of variation for yield (Environment A = 71,68, Environment B = 64,38 and Environment C = 52,07)?*

**Answer:**

We have added a brief explanation addressing this question in the Results section: “…The only exception was Yield, in which due to the particular features of the trait and the methodology used for its estimation, high CV values were estimated regardless of the environment” (please see p. 15).

Furthermore, this point has also been addressed in the Discussion section: “…The exceptions were the high Yield CVs, which may be trait-intrinsic but could also be attributable to the method used to estimate Yield, which was based on both the number of fruits per plant and the average weight of the fruits. In addition, the low mean values, especially those obtained for environments A and B, lead to higher CV values, since this measurement represents the ratio of the standard deviation to the mean, the denominator of the CV equation (Table 2).” (please see p. 24).

4. *Table 1. I suggest identifying abbreviation and acronyms at the bottom of the table.*

**Answer:**

Table 1 was moved to Supplementary material 1, following the editor’s suggestion. We have added the abbreviations and acronyms at the bottom of the table (see p. 34).

5. *Why didn't you make any mention of the parents in results and discussion since in this study, you examined 32 genotypes (page 5, line 106), consisting of a sample (n = 30) of full-sib progeny of sweet passion fruit and the two parents. Would it be possible to insert information in table 1 and table 2 using the parents as controls?*

**Answer:**

We have added parent information to the Table and included this point in the manuscript text (please see p. 14 and p. 24).

**References**

Abreu, H. K. A., Ceccon, G., Correa, A. M., Fachinelli, R., Yamamoto, E. L. M., & Teodoro, P. E. (2019). Adaptability and stability of cowpea genotypes via REML/BLUP and GGE BIPLOT. Bioscience Journal. 35(4):1071:1082.

Cullis B, Jefferson P, Thompson R, Smith AB (2014) Factor analytic and reduced animal models for the investigation of additive genotype-by-environment interaction in outcrossing plant species with application to a Pinus radiata breeding program. Theor Appl Genet 127:2193–2210.

Dias KODG, Gezan SA, Guimaraes CT, Nazarian A, Silva LC, Parentoni SN, Guimarães PEdO, Anoni CdO, Noda RW, Ribeiro CAG, Magalhaes JV, Garcia AAF, Souza JC, Guimares LJM, Pastina MM (2018) Improving accuracies of genomic predictions for drought tolerance in maize by joint modeling of additive and dominance effects in multi-environment trials. Heredity 121:24–37.

Oliveira, T. R. A., da Silva Costa, K. D., de Amaral Gravina, G., dos Santos, A. M. M., Silva, A. M. F., dos Santos, P. R., & de Carvalho Filho, J. L. S. (2018). Assessment of Resistance Status of Tomato Progenies to *Ralstonia pseudosolanacearum* and *Ralstonia solanacearum* Using Analysis of GGE Biplot and REML/BLUP. Journal of Experimental Agriculture International, 1-10.

Piepho HP, Müller BU, Jansen C (2014) Analysis of a complex trait with missing data on the component traits. Commun Biometr Crop Sci 9:26–40.

Piepho HP, Mohring J, Melchinger AE, Buchse (2008) BLUP for phenotypic selection in plant breeding and variety testing. Euphytica 161:209–228.

Resende, MDV, and R Thompson. 2004. Factor analytic multiplicative mixed models in the analysis of multiple experiments. Braz. J. Math. Stat. 22:31–52.

Smith A, Cullis BR, Thompson R (2001) Analysing variety by environment data using multiplicative mixed models and adjustment for spatial field trend. Biometrics 57:1138–1147.

Santos, A., Ceccon, G., Teodoro, P. E., Correa, A. M., Alvarez, R. C. F., Silva, J. F., Alves, V.B. (2016). Adaptability and stability of erect cowpea genotypes via REML/BLUP and GGE Biplot. Bragantia. 75: 299-306.

Santos, P., de Oliveira, T. R., Skeen, P., Nascimento, M., Danilo, K., Araújo, E. R., … Costa, A. F. (2019). GGE Biplot and REML/BLUP based-analysis of yield stability and adaptability for common beans in multi-environment trials. Revista Brasileira de Ciencias Agrarias, 14(2), 1–8.
